# Airports as spaces of dissent and protest: A spatial and historical analysis

**DOI:** 10.12688/openreseurope.18709.1

**Published:** 2026-01-09

**Authors:** Alexander Araya López

**Affiliations:** 1 Universitat Potsdam Wirtschafts- und Sozialwissenschaftliche Fakultat, Potsdam, Brandenburg, Germany

**Keywords:** airport conflicts, radical politics, civil disobedience, prefigurative politics, private jets, climate activism, police, aircraft noise, postcolonialism, mass tourism

## Abstract

Global air transport is a complex and powerful industry that contributes to economic development and international connectivity. As key infrastructure for the transport of goods and people, airports are generally considered off-limits for political dissent. By tracking protest events over the last decades, a typology of political uses of “the airport” as a space for democratic participation has been proposed, identifying its value as a site for a wide variety of protests, comprising those related to working conditions, anti-deportation, animal rights and environmental protection. In the context of the climate crisis, airports, airline companies and private jets have been under increasing criticism due to both their regional impacts (mainly aircraft noise) and the GHG emissions resulting from global air transport. Activism against new airport projects and airport expansions has become a major component of the climate movement in Europe, with 140 protest events at airports being documented and examined between 2019 and 2024. These protest events are characterized by their non-violent stance, although social movements and campaign groups have also relied on acts of civil and democratic disobedience, forms of radical politics and direct action. These protests disrupt diverse spaces of the airport, ranging from those outside the airports to more controversial acts that take place in restricted airside areas. Social movements and campaign groups appropriate spaces at the airports to increase their chances of capturing the attention of the local and global media, governmental authorities and air transport stakeholders. These protest events are subsequently shared on their own websites and social media, as a strategic form of digital activism. Activists campaigning for the protection of the planet and their local communities have also networked at the European and global levels, advocating for climate justice and a fair transition and calling into question solutions based on technological development.

## Introduction: Global air transport from the pre-pandemic to the post-pandemic world

On October 10th, 2019, visually impaired former Paralympic champion James Brown climbed onto a British Airways airplane at London City Airport and lay on top of the aircraft, denouncing the “climate impact of flying and the government’s support for airport expansion” ( Sadiq, 2019). This event took place as part of a series of protests organized by the collective Extinction Rebellion, which included activists sitting at the entrance of the airport and blocking nearby roads, two activists climbing onto the roof of the airport, and one activist refusing to sit after boarding a flight to Dublin ( Drewett, 2019). The protests captured media attention in the United Kingdom and globally.

In 2019, Greta Thunberg was becoming a major global political figure, and she was designated *Time*’s Person of the Year ( Alter *et al*., 2019), mainly because of her efforts to raise awareness about the urgency of the climate crisis, which was paired with harsh criticism regarding the emissions from flying. The term *flygskam* (flight shame) was gaining popularity in Europe and abroad, although another lesser-known term, *smygflyga* (flying in secret), was also reportedly being used by those who “wanted” or “needed” to keep flying and tried to avoid any public “shaming”, functioning as a *coping strategy* ( Becken *et al*., 2020: 15) for the incompatibility between their individual behavior and their own value systems or societal expectations regarding environmental protection.

In December 2019, a new coronavirus was identified in Wuhan, Hubei Province, China. Global health authorities considered it a serious threat. By March 2020, the COVID-19 pandemic had paralyzed the global aviation industry in an unprecedented manner, leaving airports as “emptied” spaces used almost exclusively for rescue flights and cargo of essential goods (which included a wide variety of medical supplies from respirators to face masks). According to the International Civil Aviation Organization (ICAO), for the year 2020, scheduled passenger traffic experienced an overall reduction of 2,703 million passengers (around -60%) and an estimated USD 372 billion loss in gross passenger operating revenues of airlines, compared to 2019 levels ( ICAO, 2023). The sudden reduction in capacity and travel restrictions implied a significant labor loss ( Sobieralski, 2020). During this forced impasse, environmental activists, as well as those who have been protesting unrestrained global mass tourism over the last decades, campaigned for a “green” post-pandemic future. To secure some income and to offer the consumers a way to travel safely, several airlines offered “flights to nowhere” ( Robson, 2020), mainly flights that departed and landed at the same airport, and which also included innovative services such as “speed dating” ( Coffey, 2020).

After months of lockdowns and border closures, the emergency approval of a coronavirus vaccine brought optimism regarding the return of travel and the recovery of both the tourism and the air transport industry. Since the air transport industry was decimated and perceived as a *public need*, national governments promised taxpayer-funded bailouts to save both airlines and airports ( Sorkin, 2021 for airlines in the United States; Laville, 2020 for airlines in Europe). Climate campaigners such as Greenpeace, Carbon Market Watch and Transport & Environment condemned these “blank checks” ( Keating, 2020). In the summer of 2021, with increasing numbers of vaccinated people, airports and airlines started to report “recovery” trends. Local activists feared that the comeback also meant the return of the many negative effects (or “externalities”) linked to the “normal” functioning of global air transport, from aircraft noise to road congestion. Many of these negative impacts of airports have already been studied, such as air pollution ( Hudda *et al*., 2020), water contamination ( Nunes *et al*., 2011), aircraft noise ( Flindell *et al*., 2021) and its associated health risks ( Saucy *et al*., 2021) (including risks for children ( Hygge *et al*., 2002)), and both CO _2_ and non-CO _2_ greenhouse gas emissions ( Gössling & Humpe, 2020; Lee *et al*., 2021) that contribute to aggravating the climate crisis.

This paper investigates “airports” and their surrounding areas as both the sites and objects of several protest events, and it has been structured as follows: Firstly, airports will be approached as legitimate spaces for democratic performance, considering both their local and global character and their “public” dimension. Secondly, a typology of the political uses of airports as sites of protest and dissent is presented, by historically tracking cases from all over the world. Thirdly, the diversity of spaces and strategies to protest *in* and *against* airports (including airlines and private jets) is examined, based on 140 protest events identified in Europe between 2019 and 2024. Fourthly, a brief postcolonial inquiry is included that relates to a broader discussion on climate justice and fair transition. Finally, the paper offers key remarks on the conflict between global air transport and climate activism, including some recommendations for further research.

These 140 protest events in Europe, as political communication, must be understood as crucial for practices of deliberation in democratic societies ( Habermas, 1990), since social movements and campaign groups attempt to increase their recognition and influence opinion-formation and decision-making processes. Some of these campaign groups could be considered “subaltern” ( Fraser, 1990), since they experience limited access to the public sphere and are compelled to devise strategies to overcome the inherent gaps within the democratic system. While some protesters aim to reach the media and impact political decisions through consensus, other protesters are more oriented towards forms of direct action. In this study, civil disobedience ( Celikates, 2016; Lefkowitz, 2007; Rawls, 1999) is understood as public, nonviolent, conscientious acts contrary to law that aim to foster change in the laws or policies of the government, or to influence the decisions taken by airport consortia, airline companies, the consumers, and international bodies. As Markovits (2005) points out, activists sometimes engage in disobedience to enhance the democratic process, and they might break laws that are “unrelated” to their political cause. In the case of airport conflicts, trespassing might be a necessary action to advocate for climate justice or against deportations, and it does not mean that activists are against trespassing laws. Berglund (2025) considers that disruption offers activists a form of *prefigurative legitimacy*, since disobedience and direct actions could be perceived as the “last resort” and also as a way to achieve a more democratic and just society.

## Understanding airports as sites and objects of dissent

Airports are complex spatial arrangements that fulfill several functions for both the neighboring cities and the nation-state. Their economic dimension, which is linked to the transport of people, resources and commodities, is frequently described in terms of their contribution to Gross Domestic Product and job creation. Kasarda and Lindsay (2012) published a book that centers on this economic value of airports, understanding the *aerotropolis* as the future of cities. Derudder and Witlox (2005) have highlighted the role that airports play at the global scale, for example, by interconnecting the “world city network”. At the political level, airports are important for controlling flows of people, both nationals and foreigners. For migration and tourism, airports frequently host border control operations, and they can facilitate the expulsion of those individuals who are considered a risk for the nation-state (such as the removal of diplomatic personnel in international conflicts or repatriation of “irregular” migrants or refugees with a “rejected” status). If needed, airports could be used for military purposes or to secure the needs of the nation-state, as witnessed in the essential role that these spaces played during the pandemic (particularly, for the speedy transport of vaccines and other key medical equipment such as masks or ventilators).

Boquet (2018) explored the importance that airports have for their surrounding territories in terms of economic revenue and employment, while also identifying the advantages and conflicts that airport territories create. In his examination, aircraft noise (particularly at night), unpleasant kerosene odors, and the impact on real estate dynamics, as well as environmental concerns, were identified as part of the negative effects that mobilize the local population against airports (and airlines). Faburel and Levy (2009) previously explored these airport conflicts, emphasizing the role of science and expertise of the local communities in documenting the negative effects of global air transport, a strategy that could improve their chances of being included in relevant opinion-formation and decision-making processes regarding airport infrastructure.

Airports have been designed not only to provide services to their main customers (the airlines), but to become “welcoming” spaces for both passengers and local inhabitants. As Nikolaeva (2012) has explored for Amsterdam Airport Schiphol, their design requires negotiation between multiple parties, from those involved in security to those interested in increasing the profitability of the airport. The trend of this “user-friendly” design includes a wide diversity of services, from libraries to museums to casinos. A recent example is the Felipe Ángeles International Airport in Mexico City, which includes a museum dedicated to the mammoth in its complex, after hundreds of skeletons were unearthed during the construction ( Copeland, 2022). Airport projects have also become lucrative and prestigious “opportunities” for renowned architects, with these developments becoming a form of symbolic representation of the neighboring city or the nation-state, allegedly incorporating the “essence” of the local identity in their design. Expectedly, climate activists have targeted these prominent architects, condemning their collaboration in airport projects as a violation of their environmental pledges ( McKie, 2020).

As monumental spaces, airports could be read as symbols of a nation-state identity and as spaces that contribute to shaping historical narratives. This symbolic value could be observed in the naming of these spatial arrangements, which include political figures such as Indira Gandhi or John F. Kennedy, musicians like John Lennon or Louis Armstrong, artists such as Leonardo da Vinci, soccer players such as Cristiano Ronaldo, and other local or international personalities (for example, the third international airport in Jamaica has been named after Ian Fleming, the writer of James Bond, who resided and wrote in the area ( Reuters, 2011)). Airports also appear in countless cultural products, from movies to fiction books, and their monumentality could be a product of their physical dimensions or of the affective value that these spaces have in the collective mind. In late 2020, former U.S. transportation secretary Pete Buttigieg praised the romanticism of Chicago O’Hare International Airport, pointing out that he proposed to his husband in one of the terminals ( Bonos, 2020). As a result, the airport’s account changed its then Twitter (now X) profile and self-designated as a “place of romance” ( Groppe, 2020).

As such, airports have been typically associated with their main function as spaces of transport and economic growth. However, airports have also had a political function for claim-making processes in democratic societies, although their role as spaces of dissent has been understudied. According to Parkinson (2012), there are several reasons why protesters might choose a physical location for their democratic performance. These are spaces that are accessible and visible, including spaces of power or of symbolic importance, in which a large number of people “looks like” a large number of people, and spaces that media organizations monitor on a regular basis. Do airports fulfill these criteria? The answer is dependent on many factors, which include the type of protest tactics employed by the protesters, the size and significance of the airport, the tolerance and response the respective airport authorities and law enforcement, and even the time or “when” of the protest.

Löw and Knoblauch (2021) point out that spatial conflicts are also the result of a collision between the logics of two figurations: modernity, with its emphasis on homogenization, institutionalized systems, and centralization, and the late-modern or postmodern, with its complexity of networks and mobility. To assess the challenges of contemporary protest events and the spatial transformations shaped and resulting from them, three main aspects of the refiguration of spaces are relevant: 1) *Polycontextualization* of space allows us to understand the coexistence of dissimilar spatial formations and uses. In this sense, an airport can be perceived as both a space of power and capital and as a space of destruction and environmental degradation. 2) *Mediatization*, which will be explored in detail below, elevates localized protest events to the national, supranational (or European), and global arenas, contributing to shape the discourses about the spaces where these events take place. 3) *Translocalization*, in which processes of linking and delinking create a dynamic of temporary and permanent ties (given that political decisions taken at the local scale may have significance for other places interconnected through networks). Considering protest strategies based on civil and democratic disobedience and “radical” politics, the blocking of an airport might cause significant delays and disruption, impacting not only the operations and revenue associated with these spaces but also “damaging” the dominant narratives about them, their adjacent cities, or the nation-state. Because airports are key spaces of interconnection, disruption in one airport might translate into disruption in other airports, which could contribute to capture media attention beyond the local or national level.

Endres and Senda-Cook (2011) have explored the rhetorical value of place in protests, understanding both human bodies and the chosen places as devices that could help to challenge dominant narratives and to temporarily enact alternate meanings. Bodies in an airport are expected to *occupy the space only temporarily*, for example, while boarding a plane, waiting for a connection or dropping someone off. In the case of protest events, the “occupation” of an airport requires another reading of these bodies. A symbolic red line at El Prat Airport in Barcelona ( Rocabert Maltas, 2019) or the Protestival event at Amsterdam Airport Schiphol ( Khaddari & Wiegman, 2019) are both embodied performances that challenge the ways in which airports are understood. Indeed, sit-ins or die-ins (in which protesters simulated mass dying to highlight the emergency of climate crisis) at an airport exploit the fixity of bodies as a political statement, precisely because airports are frequently associated with ideas of globality, mobility and speediness.

Routledge (2017: 16–18) explores both the spatial strategies and the sites of intervention frequently used by activists for their acts of dissent. The six spatial strategies identified in his analysis are: “ *know your place”, “make some space”, “stay mobile”, “wage war of words”, “extend your reach”* and *“feel out of place”*. In relation to airport conflicts, activists seem to rely on these strategies, not only because they have empirical knowledge of the geographies of the airport, the natural spaces and communities surrounding them, or the distribution of the negative effects associated to air transport, but because they also manage to insert themselves in these spaces, physically transforming them. The global dissent against air transport is highly mobile, with protest acts “occurring” in unexpected places and times. These social movements extend their reach through digital activism and online campaigning, while their practices challenge the meaning and functions of these spaces. In relation to the sites of intervention ( Routledge, 2017: 18–23), airports could be read as *sites of destruction* since these are the places from where the airplanes that later cause both CO _2_ and non-CO _2_ emissions or excessive noise depart and land. Airports are also *sites of consumption* and *sites of circulation*, and an activist campaigning in these spaces interferes with these dynamics, which are essential for tourism or cargo purposes. Additionally, airports could become *sites of potential*, with activists imagining alternative uses for these spatial arrangements, including closing airports and their transformation into parks.

Finally, airports can be considered *public spaces*, combining the three main interpretations of public as proposed by Parkinson (2013: 687–688): a) as openly accessible spaces, b) as spaces of common concerns, due to their use of common resources or creation of common effects, and finally, c) as spaces used for the performance of public roles, including democratic claim-making. In the first sense, airports are relatively accessible, and they are strategically designed to be reached by anyone, oftentimes interconnected with other mass transport infrastructures such as highways or rail networks. In the second sense, the impacts of global air transport are “common” by definition, either the positive impact generated by the quantity of jobs directly or indirectly linked to the industry (including the economic revenue of tourism) or other negative impacts such as aircraft noise (which could be a “common” side effect for a large number of neighbors of a given airport locally or regionally) or greenhouse gas (GHG) emissions (which could be a “common” side effect for both human and non-human living beings globally). The third sense, or the role of airports as spaces for the practice of democratic roles, will be explored in the following typology of political uses and in the section on spatial politics that focuses on recent protest events in Europe. It is necessary to emphasize that this publicness of airports is not dependent on *ownership*, given that airports could be both publicly and privately owned, managed through public-private partnerships, and part of complex global consortia with multiple stakeholders at various scales.

## Methods

To explore the long history of airports as contested political spaces as well as to examine protest events at airports in Europe between 2019 and 2024, this analysis is based on a revision of secondary studies on global air transport, a review of past and ongoing airport conflicts, an extensive search of newspaper databases on airport protests, and the tracking of several social movements and campaign groups targeting the industry in both physical and digital spaces.

Participant observation of protest events at airports was conducted in the Netherlands and Germany. Media texts were collected and reviewed for 140 protest events in Europe between 2019 and 2024, including cases in Sweden, Germany, Italy, Spain, France, Austria, Portugal, Switzerland, Albania, Denmark, the Netherlands, Belgium, Finland, Norway and the United Kingdom. Email correspondence was used to request clarification from past protest events, mainly with local campaign groups in the United Kingdom and Denmark. Some visual data was produced during the participant observation of the protest events or was requested from the respective social movements and campaign groups. Although the scope of this study is at the European level, this analysis includes four airport conflicts as main cases, namely: Berlin Brandenburg Airport in Germany, Bristol Airport in the United Kingdom, Amsterdam Airport Schiphol in the Netherlands and El Prat Barcelona Airport in Spain. The empirical information was reviewed in several languages, including French, German, Spanish, Italian, Portuguese, Dutch, and English.

In the case of Bristol Airport in the United Kingdom, Berlin Brandenburg Airport and Leipzig/Halle Airport in Germany, and Amsterdam Airport Schiphol and Eindhoven Airport in the Netherlands, several visits to these airports and their surrounding areas were conducted to observe and document the negative impacts of the industry. In the ongoing airport conflict in Southern Albania, activists provided updates about the development of the airport project and their protest events on a regular basis.

## Results

### A typology of protest events at “the airport”

Before the pandemic, some news articles identified “airports” as the “new” public arenas of social dissent. In *Curbed* ( Walker, 2017), their political value is discussed in relation to the demonstrations against the executive order signed by U.S. President Donald Trump in his first term, which prevented nationals of seven countries from entering the United States. According to this publication, more than 80 demonstrations took place across the United States, and the protests were possible because airports have moved away from the “fortress model”, becoming more “permeable spaces”. Similarly, an article in *The Economist* (2019) argued that the design of the Hong Kong International Airport, which was devised by English architect Norman Foster, functioned as an architecture of dissent, allowing the protesters to build barricades and to prevent police abuses due to the extensive system of surveillance. The piece also explained that any blockage to the airport would secure international media coverage, drawing comparisons between Hong Kong Airport’s main terminal and 19th-century Paris.

Although in recent years airports have been increasingly used and appropriated as spaces of dissent for a wide variety of local and global political issues, there is evidence that airports have always been a strategic site for (and also object of) protest events. In the following typology, five political uses of “the airport” have been identified, tracking diverse acts of dissent from the 1960s until the current post-pandemic recovery stage.

**•   Protests regarding job-related issues and work conditions (Type I)**

In this first category, the “airport” is understood as the workplace in which a given harm is committed. The motivations of these protest acts range from demanding better working conditions in terms of safety (for example, in the early years of the industry, several protest events referred to the risk of hijacking, including a 24-hour shutdown coordinated by the International Federation of Airline Pilots’ Associations in June 1972 ( Witkin, 1972)), fair remuneration and benefits (including a strike by air controllers at Heathrow Airport in London regarding a pay increase ( The New York Times, 1981)). The protesters include not only workers directly employed by airports and airlines (i.e., pilots, air controllers, flight attendants, and ground handling personnel), but also other professionals who either profit from or economically depend on airports as infrastructure (for example, taxi drivers). This type of protest is still frequent today, as in the case of a demonstration organized by taxi drivers demanding the right to access the newly inaugurated airport in Berlin ( Die Zeit, 2020), which occurred on the opening day as several climate groups were protesting against the airport.

In 2019, several protests took place in Türkiye at the site of the new Istanbul International Airport, denouncing the dangerous working conditions faced by construction workers and the reported 27 fatalities associated with this Erdoğan project ( Starr, 2018). In Mexico, transparency about the severity of injuries and deaths of construction workers was also an issue in the case of the Felipe Ángeles International Airport near Mexico City, a López Obrador project, as reported by the newspaper *El Universal* (2021).

These job-related protests are not opposed to the airport per se, and they could be considered as in synchrony with the dominant narratives about air transport, specifically economic growth and job creation. However, it is important to address that these protest events indeed use disruptive tactics to capture both media and political attention and alter dominant spatial uses of the airport when they occur.

**•   Protests regarding national and international affairs (Type II)**

This second category refers to the airport as the “stage” in which protests about “unrelated” political causes take place, ranging from domestic issues to international affairs. These protests might be motivated by the arrival or departure of a political figure or by the “symbolic” presence of an airline representing a given nation-state. In the case of political figures, a protest against Henry Kissinger in 1973 took place at the Beirut Airport. Kissinger reportedly landed at a military air base in Riyaq ( Gwertzman, 1973) to avoid the demonstration. In the case of protests directed against the foreign policy of specific countries, in 1980 ground crew refused to process the baggage of passengers traveling with Aeroflot at both Kennedy Airport and Dulles International to protest the Soviet invasion of Afghanistan ( Meyers & Pichirallo, 1980).

Similar contemporary cases are the massive pro-democracy protests at Hong Kong Airport that took place in mid-2019 ( Ramzy & Mullany, 2019) or the protests at El Prat Airport in Barcelona after Spain's Supreme Court sentenced nine Catalan leaders to prison terms of up to 13 years ( Cordero & Congostrina, 2019). In this type of protest event, campaigners rely on disruption as a strategy to communicate their political cause (and they are probably attempting to exert pressure on national authorities through the economic losses resulting from any temporary interruption of air transport movements, both passenger and cargo operations).

The airports and the airlines affected are not the object of these protest events. Indeed, airports seem to be targeted because they are strategic, but protesters do not exclusively focus on this infrastructure and might have other protest events elsewhere. This second category could also include political actions that go beyond the definition of “protest”, in the sense that they are characterized by abandoning principles of non-violence or by constituting forms of crime themselves. In recent protests for better working conditions for police officers in Haiti (similar to the aforementioned Type I regarding airport workers), which could also be described as riots, protesters broke into the airport as part of their conflict with the national government ( Aristil & Isaac, 2023). In academic literature on aircraft hijackings, the “protest” component of these criminal acts has been widely acknowledged, including instances in which hijackers distributed information about their political motives to the passengers ( Horlick, 1972).

**•   Protests regarding services provided by airports and airlines (Type III)**

The third category of protest events occurring at airports encompasses activism targeting services provided by airports and airlines, ranging from transport of certain goods to their collaboration in law enforcement. In 2010, members of the German party Die Piratenpartei staged a protest at the now-defunct Berlin Tegel Airport. The flash mob involved a small group of half-naked protesters campaigning against the use of full body scanners in the security theater ( Der Spiegel, 2010), which was perceived as an invasion of privacy. In 2013, the Bunny Alliance protested at Denver International Airport against both Delta Airlines and Air France policies regarding the transport of live animals for vivisection in labs around the world ( McGee, 2013), and similar demonstrations were organized by the collectives Gateway to Hell and Stop Huntingdon Animal Cruelty at both Manchester Airport and Heathrow Airport in 2005 ( Scrivens, 2005).

The massive demonstrations against the Executive Order 13769, informally called the “Muslim ban”, enacted by U.S. President Donald Trump in 2017 ( Chokshi & Fandos, 2017) are another example of this type of protest event, and they could be paired with other political acts aiming at disrupting “deportation flights” (including the famous case of the Stansted 15, a group of activists who locked themselves around a plane at Stansted Airport in 2017 to block the deportation of 57 people from Britain to Nigeria and Ghana ( Smoke, 2021)).

In terms of the spatiality of these protest events, the airport is more than the site where the protest is taking place, considering that there is an implicit criticism of the role that both airports and airlines play in aggravating the issues under discussion. The tactics vary from non-violent assemblies to more “radical” acts of civil and democratic disobedience (for example, forms of trespassing and even sabotage). As in Type I and Type II, these protest events are not necessarily against the airport, as they tend to be reformist in nature.

**•   Protests regarding the local and regional negative effects (“externalities”) of the global air transport industry (Type IV)**

The fourth category refers to protests about the negative effects of the “normal” functioning of the global air transport industry. Considering that there are several types of airports, ranging from cargo airports to primary hubs ( Jarach, 2001), each of these spatial arrangements impacts its surrounding environment and populations in diverse ways. For example, although activists in the Netherlands and Japan have campaigned against aircraft noise, in the Japanese context this conflict is also linked to the presence of military bases controlled by the United States ( Apter & Sawa, 1984; Lee, 2025), while in the Netherlands the aircraft are mainly for civil aviation (both transport of passengers and cargo). Local authorities frequently collect data and map these types of negative impacts, and recent research at the European level has explored potential solutions for this problem, including risk assessments ( Zaporozhets & Blyukher, 2019) and novel strategies for noise management ( Heyes *et al*., 2021).

Airport authorities and local governments tend to propose technological solutions to deal with “externalities” such as aircraft noise, for example the sponsorship of soundproofing for local schools near Heathrow Airport ( Topham, 2013). The expansion of the cargo airport in Leipzig has also sparked a series of protest events, with local activists organizing a night protest demanding the right to rest ( Lehmann, 2021). Boquet (2018: 144) has explained that the imposition of night curfews around airports led DHL to move its hub from Brussels to Leipzig, due to “growing opposition to nighttime aircraft noise”. Indeed, this type of *spatial fix* is not uncommon to tackle airport conflicts, and the relief of one community becomes the problem for another.

Both the expansion of existing airports as well as new airport infrastructure create additional issues for the local communities, including concerns about land grabbing and deforestation. In 2014, environmental activists from the Northern Forests Defence ( *Kuzey Ormanları* Savunması, KOS) campaigned for the protection of woods, lakes and sand dunes that were threatened by the new airport project for Istanbul ( Bridger, 2015; Gürtler, 2016). Similarly, the campaign Save Thano in India demanded the protection of local biodiversity and elephant corridors, after several acres of land were given to airport authorities for the expansion of Dehradun airport. The protests were the result of the planned felling of about 10,000 trees ( India Today, 2020). Other environmental issues linked to both existing airports and airport expansions include water contamination ( Sulej *et al*., 2012), light pollution, and air pollution ( Weaver & Grierson, 2016).

**•   Protests regarding climate change (Type V)**

The final category of protest events that occur at airports is related to the role that the global air transport industry plays in exacerbating the ongoing climate crisis. As mentioned above, the *flygskam* (flight shame) movement has gained thousands of followers ( Chiambaretto *et al*., 2021), and global action groups such as the network Stay Grounded ( https://stay-grounded.org/) have been formed to discuss the impacts of aviation and to campaign for a just transition, mainly inspired by theories of degrowth ( Haßler *et al*., 2019; Heuwieser, 2017). As a form of prefigurative politics ( Yates, 2020), the activists engaging in these protests aim not only to educate the public about the “real” impacts of air transport, but they also voluntarily avoid or reduce their own flying.

Indeed, the global air transport industry has become part of the targets of social movements and campaign groups advocating for effective policies against climate change, including Extinction Rebellion and Fridays for Future ( Kreil, 2021). During the pandemic, campaign groups addressing the global air transport industry were critical of the bailouts given to the industry as part of “rescue” plans, particularly because these measures were not tied to any commitments regarding the climate crisis ( Gössling & Humpe, 2020; Keating, 2020; Stay Grounded, 2020). An important characteristic of this type of protest is that it has a global scope, and although the protest might physically take place in Bristol, United Kingdom, or in Arlanda, Sweden, the main objective is to raise awareness about the “destruction” and “harm” that air transport and other carbon-intensive industries imply for human and non-human living beings. These campaigns not only center on airports, considered as “sites of destruction” ( Routledge, 2017), but also include activism against airlines and private jets. These political campaigns address the perceived (and sometimes factual) “greenwashing” of common airlines by questioning their carbon offsetting schemes (for a critique of carbon offsetting, see Watt, 2021), while also showing “skepticism” regarding other futuristic solutions based on technological development (for a discussion on techno-fixes for air transport, see Brand & Fischer, 2013; Peeters *et al*., 2016). In relation to the willingness-to-pay for carbon offsetting, Berger *et al*. (2022) analyzed over 63,000 bookings made with a European airline, identifying that passengers are largely unwilling to offset their flights, contradicting other studies that rely on hypothetical behavior. Social movements and campaign groups demand solutions that do not exclusively depend on individual will.

In both Type IV and Type V protest events, the airport is both the site and the object of dissent. However, considering the wide diversity of social movements and campaign groups, their goals, tactics, and demands for airport authorities or local and national governments tend to differ, sometimes drastically. In the case of aircraft noise, for example, some campaign groups are more reformist, demanding only a reduction of the number of flights or night curfews. In the climate movement, some campaign groups advocate for a transition to more sustainable forms of transport, and on some occasions, they demand a moratorium on airport infrastructure, a ban on private aviation and the elimination of frequent flyer incentives ( Haßler *et al*., 2019).

This proposed typology contributes to our understanding of “airports” as complex spatial arrangements, which are used, appropriated, or “disrupted” differently depending on the political motives of the protesters. While a strike by air controllers demanding fair wages is exclusively job-related (Type I), other protest events at airports are characterized by relating to several of the proposed categories. A clear example of this is the protest organized by Black Lives Matter UK activists in London, in which they temporarily “occupied” the London City Airport ( Weaver & Grierson, 2016). The protest could be read as mainly addressing a national issue, considering that it is part of a long series of acts of dissent against systemic racism and police violence against Black populations in the United Kingdom and abroad (Type II), using the airport as a “stage”. However, the activists also referred to the air pollution suffered by Black people (an “externality” of air transport, Type IV) and the role of aviation in the climate crisis (Type V). Moreover, the group has frequently highlighted the role played by airlines in deportation flights (Type III), more precisely charter flights from the United Kingdom to Jamaica. In this specific case, the multiple motives behind this protest event preclude its reduction to a single category of the typology.

### Airport conflicts, tactics and digital activism

One of the most prominent struggles against an airport has taken place in Japan since 1966 and it continues until today ( CBS Mornings, 2023). The Sanrizuka struggle, which was mainly organized by local farmers that were going to lose their lands for the planned Narita International Airport, also included the participation of student groups and Japanese New Left sects. As Apter and Sawa (1984) explain, the motives of the conflict were diverse, and they included other broader issues such as the fear of the rapid modernization of Japan and of the expanding militarism by the United States (in the context of the Vietnam War), since the airport conflict started only two decades after the tragic events of Hiroshima and Nagasaki. In terms of protest strategies, the Sanrizuka movement occupied the land with trenches, fought the police with buckets of urine and feces, destroyed the control tower and employed non-violent tactics such as selling their vegetables directly to their political sympathizers in Tokyo. The Sanrizuka struggle also weaponized children in at least one political demonstration, resulting in a confrontation between the protesting children and the police ( Apter & Sawa, 1984: 98–99). Bowen (1975) explains that the Sanrizuka conflict consisted of two stages: a first phase in which the activists campaigned following a “passive resistance” approach, relying on petitions, letter-writing campaigns, or distribution of leaflets; and a second stage of confrontation or “active resistance”, with demonstrators engaging in violence at different levels, ranging from the use of bamboo spears or Molotov cocktails to the explicit threat of physical attacks and later killing of police officers.

In Boston, a series of protest by the Mothers of Maverick Street addressed the nuisance caused by dump trucks crossing through the Jeffries Point area during the expansion of the Boston Airport (now Logan Airport), which included concerns about their children being run over by the trucks. On September 28th, 1968, the women left their houses and carried their children with them, performing a “baby carriage blockade” that forced the trucks to stop and turn around ( Vrabel, 2014: 152). The discontent with the Boston Airport was not recent, and it included campaigning against the takeover of Wood Island Park, which was given to the Massachusetts Port Authority for the expansion project. Three days after this first blockade, both women and trucks returned to the streets, only that in this occasion police used “unnecessary force” to clear the way for the trucks ( Vrabel, 2014: 153). This excessive repression motivated more citizens to join the struggle, and it forced the local authorities to declare a temporary state of emergency. After the trucks were rerouted, more protest events followed to campaign against the cutting of trees in Neptune Road, including “drive-ins” and legal demonstrations ( Vrabel, 2014: 154).

These two historical examples of airport conflicts demonstrate how complex the activism of the campaign groups and social movements is, consisting of a mixture of legitimate acts of dissent, performances, non-violent tactics, lobbying, and acts of civil and democratic disobedience. In his study about airport conflicts in Europe, Hornig (2017: 325) proposes the concept of *vertically asymmetric policies*, indicating that the negative effects or “the costs are geographically concentrated in a few local areas surrounding the infrastructure project, whereas the upper units of the political system mainly benefit from the positive effects”. Perceiving a lack of representation in the upper levels of government, the activists might indeed choose more “radical” strategies of dissent. Some contemporary protest events combine this “active” and “passive” resistance, as discussed in the next section.

In relation to non-violent tactics, one key example is the *Montagsdemonstrationen* at Frankfurt Airport in Germany ( Hornig & Bauer, 2016), which consist of a regular demonstration taking place every Monday at the Terminal 1 since 2011. The movement, however, has been active since the mid-1960s, campaigning against the excessive aircraft noise, pollution, and appropriation of forest land ( Keber *et al*., 2015; Keber *et al*., 2016). More recently, and not unlike the tactic employed by Japanese farmers, local activists campaigning against the third runway at Vienna Airport have been producing and selling bio flour in the land that would be used for the expansion. With the slogan “ *Pflugfeld statt Flugfeld*” (plow field instead of airfield), these activists symbolically connect issues of food security with the climate emergency ( System Change, Not Climate Change, 2020). In Bristol, activists from Extinction Rebellion protested the planned expansion with an aerial photograph showing activists forming the Extinction Rebellion sign, which represents a sand clock ( Grimshaw, 2019). In Barcelona, activists from Stay Grounded also created a symbolic red line at El Prat Airport, demanding air transport degrowth while highlighting issues of both environmental preservation and “touristification” ( Rocabert Maltas, 2019).

Digital activism is essential for campaigning against airports and airlines, as the case of the collective SchipholWatch in Amsterdam shows. As part of their activism against aircraft noise, SchipholWatch developed an app that allows users to estimate and collect data about the airplanes flying above their neighborhoods ( SchipholWatch, 2019). The information collected by the *ExPlane* app is also available open access for everyone, which further demonstrates the value of science, expertise and local knowledge in airport conflicts, as previously pointed out by Faburel and Levy (2009). On their website, SchipholWatch discusses current policies to regulate the industry at local, European and global levels, including reflections on the future of aviation or advice on protest strategies (for example, debating the use of stickers in the campaign targeting the airport and the legal places in which they should be placed). In 2019, the group also participated in the Protestival at Schiphol Plaza, a protest act that was coordinated by other important environmentalist groups such as Greenpeace and Extinction Rebellion Nederland ( Khaddari & Wiegman, 2019).

In Berlin, the now-dissolved campaign group Am Boden Bleiben also combined in situ demonstrations with digital activism. Among their many acts of dissent, they created a mock video claiming ownership for a series of delays that affected the new Berlin Brandenburg Airport, in which the activists disguised as penguins were portrayed sabotaging the infrastructure ( Am Boden Bleiben, 2019). A similar use of creative acts of dissent by disguised activists includes the Landing Crew, which has participated in protests by Extinction Rebellion to oppose the Bristol Airport expansion. The crew performs synchronized choreographies, while carrying aircraft landing paddles with the planet on fire and a no-airplane sign ( King & Kendall, 2020). The Landing Crew also protested outside the Wandsworth Prison in 2021, as a demonstration of support for the appeal hearing for the Paralympic champion James Brown’s case ( Sky Rebellion, 2021).

Petitions, lobbying and forms of the protest vote are also strategies frequently used by the activists. Becké *et al*. (2011) identified future changes in voting preferences among the participants of the protests against the new Berlin Brandenburg Airport, who also reported a frustration with local and national authorities after perceiving that the needs of the local inhabitants affected by the project were being overlooked. Indeed, these perceived and factual deficits in democratic institutions, which effectively limit the possibilities for participating in opinion-formation and decision-making processes, might influence activists to resort to more “extreme” acts of civil and democratic disobedience.

The aforementioned act of civil disobedience by former Paralympic athlete James Brown, who glued himself to the roof of a British Airways airplane ( Sadiq, 2019), or the protest against “greenwashing” in which nine activists of Greenpeace painted an Air France Boeing 777 green at Charles De Gaulle Airport in Paris ( Greenpeace France, 2021), are political statements that even if they “break the law”, they aim both at communicating the urgency of the issues and at denouncing the closure of democratic institutions. This type of protest strategies might be effective to capture media attention, but they could be perceived as “inappropriate” by a segment of the public. These protest events also create some security risks for airport workers, crews and passengers, and the disruption of airport operations could be exploited by local authorities, air transport representatives or journalists to discredit the protesters. Indeed, extensive literature on the *protest paradigm* describes these processes of delegitimization and demonization ( McLeod, 2007), in which news frames emphasize clashes between protesters and the police or portray the activists as foolish or ignorant, oftentimes with explicit statements from “official sources” or by exploiting witness accounts, statistics and generalizations ( Dardis, 2006). Contrary to this depiction, in their press releases, speeches and publications, social movements and campaign groups tend to exhibit substantial expertise regarding the complexity of global air transport, mass tourism, and the climate crisis, as well as a critical stance toward potential solutions for the crisis.

It is important to emphasize that the protest events that occur in physical spaces and digital activism are deeply intertwined. Some of these tactics are chosen precisely because they produce extraordinary or shocking images that are later shared on social media networks, mainstream news or the websites of the campaigning groups and social movements. The tactics are varied and cause diverse degrees of disruption, ranging from playful performances to more “radical” spatial appropriations. The protest events might also capture the attention of the users of the airports (from passengers to airport personnel), who stop to watch and request additional information or record the events with their own devices. In the following section, these spatial politics are described, and the relationship between spaces, tactics, and media is made more explicit.

### Spatial politics: Protest events in Europe from 2019 to 2024

Since 2019, protest events at airports in Europe have changed both in frequency and intensity. Each act of dissent at the airport is distinctive in terms of tactics, goals and media coverage, although some of these protest events share some similarities. In the table included as supporting data ( Araya López, 2025), it could be observed that some of these protest events involve hundreds of activists, while other actions are the result of a limited number of participants. These protest events are complex in relation to the typology, oftentimes combining environmental causes such as climate change (Type V) and aircraft noise (Type IV), with the fight for a just transition for air transport workers (Type I) or for the rights of refugees facing deportation (Type III). The table also describes how social movements and campaign groups use diverse objects of dissent ( Abrams & Gardner, 2023), including suitcases, flags, disguises, banners, signs, liquids, musical instruments, pajamas, bicycles, and glue. Some of these protest events are characterized by the participation of children, which points to climate change as a generational issue and to the health impacts caused by the local or regional environmental negative effects of the “normal” functioning of the air transport system. This spatial politics analysis does not aim to determine whether a particular type of protest is a “crime” or “vandalism”, since this might be better assessed by documenting court decisions or the tracking of fines given to the activists. It must be mentioned that even if a judge or jury considers a given protest as “illegal” or even “criminal”, this does not negate that the actions of the activists were planned as a form of protest and as a communicative act. This strategic communication might be considered as damaging for the legitimacy of the cause ( Malczok, 2021), but it is also effective in terms of capturing some media and political attention.

It must be mentioned that the data supporting this spatial analysis have been limited to protest events that happened at the airport (including inside several aircraft) or in proximity to the airport. Therefore, the data offer only an incomplete view of the political campaigning for a just global air transport system, since similar protest events do occur in other spaces. In 2019, a protest event in opposition to the Montijo Airport, a new airport project proposed near Lisbon in Portugal (now “on hold”), consisted of a performance that interrupted the speech of Portuguese Prime Minister António Costa, with activists from the group Aterra throwing paper planes ( Correia, 2019) from the stage of the Feira Internacional de Lisboa. Francisco Pedro, an activist who tried to reach the microphone to deliver a speech, faced a lengthy legal process after being charged with “qualified disobedience” ( Público, 2025). In 2022, on the opening day of the Laver Cup in London, an activist from the group Sacrifice for Survival interrupted the tennis match by entering the court and setting his arm on fire, while wearing a white t-shirt with the message “End UK Private Jets” ( Carayol, 2022). These two cases exemplify activism targeting air transport that takes place in other spaces where media attention is anticipated.

In the case of the resistance to the expansion of Bristol Airport, several protests took place at College Green, outside the Bristol City Council as well as outside Bristol Civil Justice Centre, for example after the announcement that the legal challenge was dismissed and that the planned expansion was granted, with activists covering themselves in fake “blood” ( Bristol 24/7, 2023). XR Youth Bristol also campaigned against the expansion near the central train station, specifically by temporarily blocking the bus that transports both workers and passengers to Bristol Airport ( XR Youth Bristol, 2022) on at least two occasions. Another protest against the Bristol Airport expansion, which happened at Weston-super-Mare, and included over 100 activists burying their heads in the sand with a giant ostrich ( Brock, 2020) besides them, was extensively covered by local and national media, since some decisions on the project were taking place in the area. In 2024, the march The Big One took place in London demanding real climate action and the end of airport expansions. The march included activists from Bristol, as well as protesters from other airport conflicts in the United Kingdom, among other groups such as aviation workers who now advocate for climate protection ( Safe Landing, 2023). For analytical purposes, these protests have been excluded from the supporting data, even when they are extremely important when examining each separate case.

Consequently, the airport should be considered as a space characterized by its porous boundaries, since it does not stop at the fence. As an open system, passengers, supplies (such as jet fuel) and merchandise enter the airport, as aircraft, solid waste and cargo goods leave it. In the case of aircraft noise and GHG emissions (not only from the airport but from the flights), these outcomes of the air transport industry are not restricted to the physicality of airport. These “externalities” occur in movement, reaching multiple spaces. The decisions about the airport and their functioning are also taken in multiple sites, and while they might be more visible in proximity of or at the airport, other decisions about the airport are taken in far-removed locations.

The 140 protest events documented and analyzed in Europe between 2019 and 2024 ( Araya López, 2025) use diverse spatial strategies, including protest in proximity to the airport, as well as in the departure halls, landing lanes, frequent flyer lounges, and inside several aircraft. This number is non-exhaustive, considering that other protest events at European airports might have been omitted because they had limited media coverage (when reported exclusively in local media) or due to language barriers. Moreover, since protest events at airports have continued in 2025, the supplementary data has limitations. The spatial politics of these protest events are examined in the following subsections:

**•   Before the airport**

Most of the cases identified in this analysis are airport conflicts in which the infrastructure has already been built and operating for a long time. The case of the new airport proposed in Vjosa Narta, in Albania, is a case in which resistance precedes the physical airport. The airport, currently under construction, is located near a national park and bird reserve, with activists claiming that the boundaries of this protected natural area were redrawn to enable the controversial project ( Peeters, 2022). The reserve is currently inhabited by many species, including flamingos, pelicans, and other birds ( Gale-Coleman, 2023; Peeters, 2022). The airport has been justified to boost tourism in Southern Albania and there are parallel projects that would benefit from its construction. Hotel development in the region is also being contested, specifically two luxury real-estate projects proposed by Jared Kushner and Ivanka Trump ( Lipton & Karaj, 2024). Local resistance has mobilized against these projects through both demonstrations ( Goga, 2023) and legal procedures, and activists have appealed to European authorities to intervene, given Albania’s aspiration to join the bloc. From a more-than-human perspective on this spatial conflict, it could be argued that the lives of flamingos, pelicans, and other threatened species are treated as abject ( Fleischmann, 2023), as nonhuman beings that can be “sacrificed” for both critical infrastructure and tourism development. In the protest events, activists from Protection and Preservation of Natural Environment in Albania (PPNEA) carried banners and signs depicting the threatened bird species, and their demonstration included live music ( Goga, 2023).

The protection of the natural environment and the species inhabiting the area designated for the airport is an issue that became central in a major airport conflict in France, which indeed ended with the official cancellation of the project. The proposed airport for Notre-Dame-des-Landes was challenged by local residents and various organizations for years, with activists occupying the area in which the construction works were planned. In their book entitled “We are nature defending itself” ( Fremeaux & Jordan, 2021), two activists explain how the occupation evolved and how they organized to fight the national government, while also creating a community that was less invasive regarding the surrounding environment. The activists relied on media and lobbying to communicate their cause to the French public, and after years of confrontation and police abuse (particularly during eviction operations), the national government was forced to concede the plan and to legally recognize the rights of the defenders. Indeed, the Zone To Defend (or ZAD, Zone à Défendre in French) has become one of the most successful chapters in the resistance to airport infrastructure in Europe and globally.

**•   In proximity to the airport**

These protest events are characterized by occurring in relative proximity to the airport, frequently by disrupting the systems that allow these spaces to function. In the case of Letzte Generation in Germany, highways leading to major airports such as Berlin Brandenburg Airport have been targeted by the activists ( Der Spiegel, 2022). The actions by XR Youth Bristol targeting the public service transport to Bristol Airport are another example of this logic, although the distance between the protest and the airport was significantly greater. A particularly interesting case has been tracked in Sweden, in which activists from Flyglarm Arlanda and the local Extinction Rebellion chapter successfully blocked the trains delivering jet fuel to Arlanda Airport, on at least two occasions ( Stay Grounded, 2019).

Some of the protest events include marches that move towards the airport, as was the case of a massive demonstration that took place in Nice in 2023, with over 400 activists carrying signs and banners along the Promenade des Anglais, to protest the planned expansion of Nice Côte d'Azur Airport ( Blache, 2023). In the United Kingdom, a pilgrimage organized by Christian Climate Action also targeted the projected expansion of Bristol Airport, with activists walking from the Bristol Cathedral to the main terminal. The activists did some symbolic stops at local churches and temporarily entered the terminal to demand respect for the planet ( Wimperis, 2023). Police authorities oftentimes intervene to prevent protesters from reaching the airport after they have announced a protest, as it happened in Berlin Tegel Airport in 2019 ( Peter, 2019).

**•   Outside the airport**

Several protest events against planned expansions, private jets, aircraft noise, and demanding “real” action for the climate crisis take place outside the airport (mainly the terminals, but some protests also occur near the fences). In the case of Bristol Airport, activists have protested in the nearby A38 roundabout that leads to the airport ( Eyes On Bristol Airport, 2023), since this area is the closest point to the main terminal that is still within “public space”. In Barcelona, Greenpeace España advocated for a transition to railways with a creative installation near a viewpoint of El Prat Airport ( El Periódico, 2021; Greenpeace España, 2021), at a time when the public debate about the potential expansion intensified in both mass media and the political sphere. In Copenhagen, activists have also chosen to demonstrate outside the airport, mainly because blocking the road to the terminal involves minimal legal consequences. These demonstrations have been organized on a monthly basis ( Scientist Rebellion, 2024).

These protests use diverse tactics, from blocking nearby roads to sharing information with the users of these spaces. In protest events at both Copenhagen Airport ( Extinction Rebellion Danmark, 2024) in Denmark and Maastricht Airport ( RTV Maastricht, 2024) in the Netherlands, activists have shared flyers with the public. In 2023 in Sweden, activists from Scientist Rebellion posted academic papers on the façade of the Grafair Jet Center, and later coated the terminal in red paint, as part of their disruptive activism against private jets ( Extinction Rebellion Sverige, 2023). On November 5th, 2022, hundreds of activists from diverse groups campaigned outside Amsterdam Airport Schiphol, marching around the airport complex while holding banners and signs. This protest event included a sit-in inside the building, with activists chanting and clapping. Simultaneously, a group of activists rode their bikes on the tarmac and blocked private aircraft that were parked at the airport ( Ketcham & Komanoff, 2022). This case demonstrates how diverse spaces can be targeted with a variety of tactics during the same “protest event”, allowing activists to support the cause in the ways that they feel more comfortable. In protest events as complex as this, the message of the activists is less easy to ignore for the passengers, airport authorities, and media organizations.

**•   Inside the airport (landside)**

Protest events inside the airport are not uncommon and they are varied in terms of spatial tactics and disruption. In Amsterdam Airport Schiphol, sit-ins and performances by Extinction Rebellion and the Red Rebels have been documented, but also there have been blockades of the frequent flyer lounges ( van Zoelen, 2024) as well as pouring of water to represent the flash flooding and human loss in cities like Valencia ( Extinction Rebellion Nederland, 2024), which has been considered a weather event intensified by climate change. At Heathrow Airport in 2024, two Just Stop Oil activists, including Phoebe Plummer, who was involved in a protest at the National Gallery in London in which canned soup was thrown at van Gogh’s Sunflowers ( Araya López & Davis, 2024; Rufo, 2024), sprayed orange paint on the windows at Terminal 5 and on several flight information boards, with the use of fire extinguishers ( Patrick, 2024). This action was part of the Oil Kills campaign, which took place in the summer of 2024. As social movements and campaign groups have realized, coordination of multiple protests as a “wave” increases their chances to attract both media and political attention and therefore protest events that take place simultaneously in several airports, including across borders, are a strategic approach. In Vienna, the group Letzte Generation Österreich participated in the campaign, by spilling orange paint on the floor in one first protest and by throwing confetti from the second floor of the terminal, holding signs with the phrase “Oil Kills” in German and blocking some areas with a sit-in some days later ( Letzte Generation Österreich, 2024). Activists also delayed a flight from Vienna to Rome, by refusing to sit while onboard and giving a speech ( Kronen Zeitung, 2024).

Other protests inside the airport use more playful, creative and performative tactics. At Bristol Airport, activists from XR Youth Bristol performed a die-in to object the planned expansion and the GHG emissions of air transport. Around 30 activists participated in the protest, which consisted of bodies covered with white sheets and some activists wearing hazmat suits and face masks ( Jenkins, 2022). In France, activists organized a pajama party at the terminal at Nantes Airport to protest both climate change and aircraft noise, advocating for the right to sleep. The protest included bodies on the floor sleeping, cuddling with stuffed animals. Other activists representing airplanes were “overflying” those trying to sleep ( AFP, 2022). In both protest events, the permanency of the bodies counters the spatial logic of the airport, which is tied to discourses of mobility and speed. By refiguring spatial uses and the narratives about these spaces, activists not only re-signify the airport, but make visible the “externalities” linked to air transport as a global industry. Other spatial appropriations include displaying banners, playing music, projecting videos, and even polling passengers’ opinion regarding airport expansions plans (for this action by Extinction Rebellion at Gatwick Airport in London, see Pole, 2024)

**•   Inside the airport (airside)**

As mentioned before in the case of Amsterdam Airport Schiphol, activists normally demonstrate in diverse sites surrounding and within the airports. Some of these protest events could be read as a nuisance or a disruption, but the actions that take place inside the airport in the airside areas are the most controversial. While discourses of securitization and risk are frequently (over)used to question the legitimacy of dissent, protest events in the airside areas imply some level of risk to both airport users and the activists. Some of the potential charges include trespassing and property damage, but oftentimes activists are also threatened with terrorism charges. Regarding the risk of the protesters, a sort of *legal paternalism* has been identified in the debate about the legitimacy of these protest events, which often includes “advice” on how to protest, where and which tactics should be preferred. If these protest events are considered as acts of civil and democratic disobedience, it is evident that the activists are aware of the risks of engaging in this type of radical protest, which points to their willingness to accept the potential punishment for their transgressions.

The first protest event mentioned in this paper, the case of activist James Brown, who lay on top of a British Airways aircraft ( Sadiq, 2019), is a clear example of actions in the airside areas. As mentioned above, in Amsterdam Airport Schiphol, activists rode bikes and blocked private jets ( Extinction Rebellion Nederland, 2022; Ketcham & Komanoff, 2022). In 2024 in Germany, activists from Letzte Generation glued themselves to the landing lanes in coordinated actions at several airports, namely Berlin Brandenburg Airport, Nuremberg Airport, Cologne/Bonn Airport and Stuttgart Airport ( Connolly, 2024). In another protest, Letzte Generation activist symbolically planted trees in the tarmac ( Hessenschau, 2024), which could be interpreted as a form of prefigurative politics regarding reforestation. The performance with small trees on pots, half-covered in soil and with the activists dressed as gardeners carrying shovels uses humor to address serious environmental issues linked to airport expansions. The felling of trees has been contested in many airport conflicts in the United States, India, Türkiye and Albania. In a protest against the expansion of Leipzig/Halle Airport, which is the world’s largest DHL hub, activists also symbolically planted trees on the construction site outside the airport, as part of a massive demonstration and march in July 2022 ( Freitag, 2022).

Other protest events that occur in the airside areas have targeted the airlines, private jet companies and aircrafts. As mentioned above, nine Greenpeace activists coated an Air France Boeing 777 in green at Charles De Gaulle Airport in Paris ( Greenpeace France, 2021), an action that denounced the “greenwashing” of both the air transport industry and the national government, with activists demanding an abandonment of several airport expansions projects ( Euronews, 2021). In 2020 in Sweden, an activist wearing a face mask and a green cape with the Extinction Rebellion symbol, glued herself to a private aircraft parked at the Ängelholm/Helsingborg Airport ( Johansson, 2020).

Activists have also refused to sit while onboard and given speeches to the passengers. This tactic has been documented in the United Kingdom ( Drewett, 2019), Austria ( Kronen Zeitung, 2024) and more recently in Portugal ( Climáximo, 2025).

### The postcolonial question

Before the pandemic, a podcast by the BBC World Business Report (2019) discussed the development of aviation in Africa, which explored political issues such as the creation of a common “open skies” initiative between African nations and the challenges to operate low-cost airlines. The value of expanding air transport on the continent was linked to needed economic growth, job creation and the enhancement of transport infrastructure. This discussion took place while several social movements were campaigning for air transport degrowth in Europe. In a discussion paper, the network Stay Grounded and the UK Trade Union PCS have emphasized the need of fostering a just transition ( Stay Grounded, 2021a), considering issues of postcolonialism and dependency while devising potential alternatives for the workers of the affected industries.

In the context of the pandemic, impoverished countries dependent on tourism in the Global South suffered significant decline in their Gross Domestic Product (GDP), including various Small Islands and Developing States (SIDS) ( Gössling & Schweiggart, 2022: 2), since these countries rely on air transport to connect with the world. Although in several regions of the world there are significant challenges for creating alternative infrastructure to substitute air transport, this does not mean that new airports or airport expansions are uncontested. These airport conflicts are slightly different from the resistance in the European context, although they share some commonalities.

In Peru, a new airport project (currently under construction) in the town Chinchero, near Machu Picchu, raised concerns due to its potential impact on the Inca ruins, as well as environmental issues such as noise and air pollution ( Collyns, 2019). The opposition to the project was generally expressed as public debate in local media and lobbying with politicians. Local communities have demanded from the national government the prompt conclusion of the project, threatening to protest and occupy Machu Picchu, after major delays ( Angulo, 2025). This situation is not uncommon in communities highly dependent on tourism flows, with one segment of the local population advocating for environmental protection while another is concerned with the potential economic benefits (for the case of cruise tourism in Venice, see Araya López, 2022).

In Uganda, another conflict emerged after the planned expansion of the airport in Kasese. Local communities, particularly women, have advocated for the preservation of natural spaces, also demanding the protection of wildlife in Queen Elizabeth National Park, which is home to thousands of African elephants. Issues of land grabbing, land acquisition, displacement and fair compensation have also been highlighted by local activists ( Stay Grounded, 2023). Ittner *et al*. (2025) have explored issues of contested land in airport conflicts in Asia and Africa, focusing on cases from Nepal, Sri Lanka, India, Indonesia, Côte d’Ivoire, South Africa, and Kenya.

In 2025, the Indian government announced their plan to boost air transport, aiming to build 350 airports by 2047 ( The Economic Times, 2025a), and opening a new airport every 50 days ( The Economic Times, 2025b). Ongoing airport projects have been resisted by local communities, with both peaceful tactics and more radical forms of dissent. In Uttar Pradesh, local farmers demanding the protection of their land protested the expansion plans for the Azamgarh Airport, with activists organizing a massive march ( Rizvi, 2023) and black flag agitations. A local campaigner, depressed by the potential loss of ancestral land and home, allegedly suffered a heart attack ( Jafri, 2022), becoming a martyr for the Makan Bachao Khet Bachao (Save the houses, save the fields) movement. More recently, local communities outraged by a road closure needed for the expansion of Kolhapur Airport, that forces them to take a detour of 20-25 km, protested their displacement with a march and by blocking access to the airport ( Free Press Journal, 2025). The campaigners also threatened to demolish the wall that prevents them from accessing the road, unless an alternative route is provided.

Although these airport conflicts are mainly related to land issues and the protection of heritage and the local ways of life, the environmental impact of this new infrastructure is still concerning, particularly in relation to climate change. It must be mentioned that these types of conflicts do not emerge only in relation to air transport, since port infrastructure, highway and railway projects also imply struggles regarding land grabbing, displacement and segregation. International collaboration has been fostered to track these conflicts all over the world, including online tools such as the Global Atlas of Environmental Justice ( https://ejatlas.org/).

In Brazil, over 2000 families of Vila Nazaré were threatened with forced eviction to expand the Porto Alegre–Salgado Filho International Airport, which is managed by the German operator Fraport. In Europe, the network Stay Grounded (2021b) and the German collective Am Boden Bleiben (2020) campaigned against the eviction, addressing the unfairness of global air transport while denouncing human rights violations. Local activists from Vila Nazaré were allegedly met with severe police violence, as well as criminalization of their protest actions. This international collaboration is relevant considering power disparities and historical oppression, particularly since German companies and governments were involved in the conflict, and European activists campaigned from within their area of influence. The collaboration also evidences how complex and intertwined global air transport is, and it connects the activism against aircraft noise in Frankfurt Airport with the activism against land dispossession in Vila Nazaré. Stay Grounded has also organized webinars and conferences with the participation of local activists from Mexico, Dominican Republic and Colombia.

For many nations and regions in the Global South, new airport infrastructure and airport expansion is key to securing economic development and creating jobs that contribute to the well-being of their local population. These countries and regions oftentimes face significant challenges in building alternative, “greener” infrastructure such as highways or railways, considering issues of political instability, geography, lack of funding and political will, and dependency on global tourism. This creates an insurmountable dichotomy: either these countries and regions continue to invest in air transport and further exacerbate the climate crisis, or wealthy nations and corporations that have historically emitted more should facilitate their transition towards less carbon-intensive forms of transport. Airport conflicts in the Global South are also the result of capitalist logics of connection and disconnection that characterize the complex power-geometry ( Massey, 1991) of global air transport and tourism, and this might explain why climate activists in Europe are networking with social movements and campaign groups in these regions and countries.

## Discussion

Although airports are functionally planned and developed mainly for the transport of goods, resources and people, and to boost economic growth for the cities and countries in which they are located, there is extensive evidence that these spatial configurations also have a political value as sites of democratic claim-making. Historically, airports have been the “stage” in which protests regarding job-related issues, national and international affairs, and controversial services provided by both airports and airlines are contested. In other protest events, airports are both the site and object of dissent, mainly when these political actions relate to the local and regional negative impacts of the airports, as well as to their role in aggravating the climate crisis. The intensity and frequency of the spatial interventions vary according to the social movements or campaign groups, and some of these protest events are characterized by a complex merging of political causes.

In the 140 protest events documented and examined at European airports between 2019 and 2024, the degree of spatial appropriation is highly variable, with protesters campaigning in proximity to the airport as well as in restricted airside areas. The tactics employed by the social movements and campaign groups seem to respond to multifaceted criteria such as innovation and creativity, the expected punishment for their actions, and primarily the possibility to secure media attention or to influence both local, national authorities and key stakeholders in the air transport industry. In the academic literature, airport conflicts and protest events at airports have been generally explored as isolated cases, which do not effectively depict how extensive the criticism of global air transport really is. Europe is distinctly the region in which resistance to new airports, airport expansions and the inequality of air transport is more salient, including protest events that occur either simultaneously or in “waves”, as well as across borders. Some of these protest events are also an expression of international solidarity, highlighting the plight of vulnerable communities in the Global South and connecting their struggle with local and regional negative impacts of air transport in Europe.

Each of the protest events and airport conflicts listed in the supporting data requires a more detailed analysis regarding the goals of the protests, the “success/failure” of the actions, the legal or economic consequences for the activists, and the media coverage that resulted from the events. In the case of goals, the political communication seems to be oriented towards key stakeholders in the air transport industry and the local or national government. The users of airports and the general public seem to be a supplementary target audience, but the protest events tend to demand structural and systemic changes that are independent of individual preferences, focusing on those with the power to enact systemic transformations. In the case of private jets, the demand for a ban should not be reduced to a personal attack on those who own such aircraft or hire charter services, but it should be understood as a request to address wealth inequality and restrict the high-carbon lifestyles of the super-rich.

Determining the success or failure of each protest event, or of the social movements in which these actions are grounded, is a speculative exercise. Firstly, some campaign groups and social movements define success in diverse terms, from delaying the planned projects to ending their protest events with minimal costs (e.g. arrests, fines, confiscated equipment, injuries, etc.). Secondly, if protest events are assessed as isolated events, their impact might be insignificant as compared to the power of the global air transport industry. However, if these protest events are considered as cumulative, the sum of all these protest events could actually influence decisions taken by relevant stakeholders, including local and national governments or airports and airline representatives. Thirdly, regarding the political communication goal of these protest events, their message about the negative impacts of air transport and their criticism of dominant narratives about the industry is effectively transferred, which could strengthen the movement by securing new members and donations or by contributing to educating the national and global public about the issues.

It must be mentioned that these efforts to challenge dominant narratives regarding global air transport occur under extremely unequal circumstances compared to the resources available to both airport and airline corporations and national governments. The public perception of airports is generally positive, and complex negative impacts such as aircraft noise or GHG emissions are presented as problems that could be solved with technological advancements. To those not directly affected by the “externalities” of air transport, airports are spaces that are used only temporarily, and some relative ignorance of the “public” regarding their functioning and their contested history is expected. Since airports and airlines are intertwined with the global tourism industry, their advertising is tied to ideas of travel, leisure and exoticism, and their public image frequently highlights their “efforts” to commit to sustainability. While some airports claim to have achieved “carbon neutrality”, even if the accreditation process has been called into question by scholars and campaigning groups, the GHG emissions of flights are rarely included in the inventory. From the perspective of the activists, the “real” solutions to many issues linked to air transport are a substantial airport degrowth, a moratorium on new airport infrastructure, the elimination of frequent-flyer benefits, a ban on private jets and progressive taxation for frequent-flyers. As Brand and Fischer (2013) have explained, these dissimilar views have created a split in environmental discourse, in which “technophilic” and “technophobic” statements compete with each other.

The legal and economic consequences for protesters are also diverse, and they are dependent on the societal tolerance for dissent. As Whitehead (2021) indicates, the citizens, the State and the activists are involved in a constant process of negotiating the limits of the legitimacy of protest. This might help explain why some protest events are no longer considered as “legitimate” or why more radical politics might become “normalized”. In Germany, activists have been asked to repay the costs of the police operation needed to respond to their protest events, which in the case of Letzte Generation amounted to 6,400 Euro for their actions at Berlin Brandenburg Airport ( RBB24, 2024). This strategy has been adopted to discourage similar actions at airports. Ten activists from the Letzte Generation have also been asked to compensate German airline companies for 400,000 Euro for their protest event at Hamburg Airport ( Knödler, 2025), which triggered the cancellation of flights because activists were glued to the tarmac. The German government has also proposed changes to the law to punish activists with prison terms and heavy fines for their actions at airports ( Bundesministerium des Innern, 2024). In 2022, the Airport Council International published advice for airports regarding how to deal with increasing dissent at airports and proposing some strategies for mitigation ( ACI, 2022). In the United Kingdom ( Tobin, 2024) and the Netherlands, injunctions have been used to keep activists away from airports, which prompted the collective Extinction Rebellion Nederland to legally challenge the ban against 37 activists ( NL Times, 2025), who were prohibited from accessing secured parts of Schiphol Airport for five to ten years. In Spain, activists from Futuro Vegetal have been equally threatened with fines and imprisonment for their protest events at Ibiza Airport ( Ribas, 2025).

In Denmark, activists from the Scientist Rebellion chapter used airport carts to “slow down” the revolving doors at Copenhagen Airport, while other activists distributed flyers to passers-by. Airport security responded by blocking the door, so the activists sat on the floor outside the terminal. Activists were wearing hi-vis vests with statements demanding a ban on private jets, as well as banners and signs ( Scientist Rebellion Denmark, 2024a). In response, airport security improvised a sort of “tent” around the activists. This type of policing has been employed in other protests related to climate activism, including actions targeting artworks at museums and art galleries ( Araya López & Davis, 2024). By restricting visibility, the potential impact of the protest event is limited, and the message of the protesters is suppressed. These visual barriers not only prevent activists from engaging with members of the public who might be interested in their political cause but also obstruct the recording of the event in photographs or videos, which social movements and campaign groups frequently share in their social media channels and are oftentimes published in mainstream media.

In short, activists campaigning for a cause are at a disadvantage regarding their political influence and possibilities to reach the national or global public. Aiming to influence both opinion-formation and decision-making processes, the protesters are compelled to choose tactics that oftentimes fail to capture significant media attention. Only a few of the 140 events included in the supporting data reached major international media, and most of the actions were only reported in local or national media (which respond to diverse newsworthiness criteria compared to those prioritized by major global news organizations). Some of the protest events have only been documented and shared by the groups themselves, mainly in their official social media accounts or websites. More radical politics such as acts of both civil disobedience and direct action seem to secure more media attention, particularly when protests are coordinated in “waves” or occur simultaneously at various airports.

Another feature of the 140 protest events and one that has been understudied is the aesthetics of each political act. While activists frequently carry banners and signs communicating their demands, their protest events oftentimes defy dominant narratives about airports in various ways. Spilling liquids that resemble oil, blood or floodwater directly connect the airport to the sites in which destruction is happening. Similarly, die-ins and pajama parties create an aesthetic in which the body is presented as vulnerable, with its vital cycles disturbed by the power of this global industry. In the case of Extinction Rebellion and Scientist Rebellion Denmark (2024b), activists portrayed some of the wealthiest users of private jets in Denmark as bloodsucking vampires in a Halloween-themed protest, challenging their status and using humor to criticize their high-carbon lifestyles. In his research on artistic transgressions, Neufeld (2015) has proposed the concept of aesthetic disobedience, which could be adjusted here to refer to political acts that challenge “dominant aesthetics” such as the pristine environment of the airport, by exposing the pollution and negative impacts on both human and non-human lives or by imagining alternative uses of this space (for example, as a park or farmland).

In terms of spatial politics, the question remains whether activists are freely choosing the airports as the sites of their protest events or are *being forced* to demonstrate precisely at these locations because they are the spaces in which the social and environmental harms are caused. As mentioned above, airport conflicts go beyond protest events at airports and they include political actions elsewhere, mainly at sites where decisions regarding global air transport are taken. While the protests are perceived as a nuisance and disruption by a segment of the public, airport and airline stakeholders, or local and national authorities, the importance of the protest for improving the quality of the public debate on issues such as climate change or deportation tends to be overlooked or minimized. As discussed in key studies on civil disobedience, activists might be morally obligated ( Lefkowitz, 2007) to engage in political dissent at the airport, especially when their inner values prioritize the preservation of nature and life (human and non-human) over more materialistic concerns (such as job creation or economic growth).

## Conclusions

Airports, usually defined as key infrastructure for economic development and international connectivity, have historically been used as spaces for political claim-making on a wide variety of causes, although they have been generally labeled as “off-limits” regarding protest and dissent. The spatial interventions produced by protesters at airports vary from peaceful gatherings in their proximity to more disruptive tactics that aim to alter the “normal” functioning of this global industry. While airports do not necessarily reach the level of “the square” or “the street” as sites for democratic performance, the disruption and appropriation of these spaces contribute to enhancing public deliberation on societal issues, mainly because both national authorities and media organizations – and to some extent the public – care about what happens at airports.

Over the last decade, airport conflicts have intensified in Europe, predominantly due to concerns about climate change, but also because of the negative impacts of air transport and mass tourism on the neighboring cities and regions (including spaces of nature). For many campaign groups and social movements, airports are spaces of harm and destruction, symbols of unequal consumption and legitimate targets of dissent. Between 2019 and 2024, 140 protest events at airports in Europe were identified, and the coordination of actions at the supranational level has become a strategic way to ensure media coverage. Indeed, media organizations and even academic studies have primarily reported these conflicts as “isolated” or by comparing cases at the national level, and few attempts have been made to understand the systemic impacts of the air transport industry in Europe or globally. This study has tracked resistance against air transport development in Germany, the United Kingdom, France, Spain, Portugal, Belgium, the Netherlands, Austria, Italy, Denmark, Sweden, Finland, Norway, Switzerland, and Albania. For social movements and campaign groups, the negative effects of global air transport are not exceptional, since aircraft noise, GHG emissions, deforestation and other social and environmental problems are common across diverse airport conflicts. In the case of the Oil Kills campaign in the summer of 2024, the coordinated actions included protest events in Boston, United States, and Montreal, Canada. This spatial and historical analysis could be expanded to incorporate activism and resistance concerning airport conflicts in wealthy nations outside Europe.

It is expected that protests at airports will continue in the upcoming years, particularly as the climate crisis continues to worsen. National governments and airport stakeholders have been advocating for harsher punishments for protesters at airports, and activists have been decrying a perceived criminalization of dissent. While there is some evidence that some criminalization is occurring, more research is needed to examine how effective these proposed deterrence policies are and what the actual punishment imposed on the activists is. In relation to the role of the police, there is an urgent need to investigate claims of police abuse that have been reported by both activists and media articles, including accusations of illegal surveillance, infiltration, and extrajudicial punishments. Similarly, as in the case of activists Phoebe Plummer, Francisco Pedro and James Brown, more research is recommended to explore how these campaigners are portrayed by mainstream media organizations, and whether “personal attacks” are strategically used to discredit the climate movement as a whole.

In relation to dominant narratives, an extensive analysis of media texts about protests at airports could offer important insights on how the global public learns about these events, and whether any delegitimization of the listed protest events is taking place. This study did not include the perspective of airport authorities regarding dissent at the airport, primarily because the four main cases refused to participate in the research, claimed to have limited availability for interviews, or ignored the email correspondence. New research could assess the perception of protests and dissent among various airport stakeholders, as well as among the general public, from those affected by the protest events to those who learn about them in the news or on social media.

## Ethics and consent

This spatial and historical analysis of the political uses of airports in Europe has been predominantly based on *public information* collected from various media outlets, archives, reports and secondary literature, as well as social networks (including YouTube, X (formerly Twitter) and Instagram) and the websites of social movements and campaign groups involved in activism for climate justice and a fair transition of the global air transport system. The ethics guidelines for the PROTEST-AIRT project were approved by the respective Ethics Committee at Universität Potsdam on April 17, 2023 (No. 65/2022). Consent was not required for this spatial and historical analysis, since no sensitive data of specific individuals were collected. However, protective measures were taken to minimize risk and avoid harm to any individual mentioned in the media texts. Social media data were restricted to the official accounts of campaign groups and social movements.

## Data Availability

Zenodo: PROTEST-AIRT - Political acts of dissent at airports in Europe (2019–2024) [Data set]. https://doi.org/10.5281/zenodo.15299010 Araya López (2025). This project contains the following underlying data: README v3.pdf Table - Protest acts at airports 2019–2024 v3.csv Table - Protest acts at airports 2019–2024 v3.pdf Data are available under the terms of the Creative Commons Attribution 4.0 International license (CC-BY 4.0).
