# Supplementary material for: Airports as spaces of dissent and protest: A spatial and historical analysis
Source: Open Res Eur. 2026 Jan 9;6:20. [Version 1] doi: 10.12688/openreseurope.18709.1 (PMC13054253; doi:10.12688/openreseurope.18709.1)
Supplement: Peer review history

# Reviewer report: Reviewer response for version 1

**DOI:** 10.21956/openreseurope.20239.r71317

**Published:** 2026-04-22

**Authors:** Clare Saunders

**Affiliations:** 1 a Politics Unit, Department of Humanities and Social Science, Cornwall,, University of Exeter, Cornwall, England, UK

This is a very interesting paper that presents a useful typology of political uses of “the airport” as a space for democratic participation. It finds four types of democratic participation at (and around) airports related to job-related issues, national and international affairs, airport services, negative (not climate-related) externalities, and climate change.

 The abstract mentions that airports are sites of variety of struggles: working conditions, anti-deportation, animal rights among them. I would suggest a tighter correspondence between the abstract and the typology of five types of airport protest.

 The paper needs to be a bit more clearly written so that it sets up airports as general sites for democratic participation. Although struggles on a variety of causes are mentioned, there are times throughout the piece (including in the abstract) where the article gives more emphasis to climate change than the other issues. At times the paper reads as if the story is almost exclusively about climate change protest, but then the analysis brings us back to the broader issues.

 The functions of an airport are clearly introduced. However, more could be done to link those features (i.e. national pride, translocation, polycontextualization, mediatization etc.) to the nature of airport protest. For example, how does the work of Routledge (who identify the six spatial strategies used by activists in sites of defence) specifically fit in with the characteristics of airports in contrast to other (e.g. less translocated and polycontextualised) spaces, such as an occupation of the streets. The literature and the analysis, in general, should talk to each other much more coherently.

 Towards the end of the review of the literature, I would recommend a slightly more nuanced discussion of airports as public spaces. The parts that are open to the public are very different from the parts that are open only to ticket-holders who have passed through security. This could be linked in with the results section about spatialities (i.e. protests inside and outside of airports). And what does the spatiality (public versus passengers) mean about the nature of protests that happen, and, more specifically, in relation to their role in democracies?  There are some interesting debates around democracy and social justice that might need to be at least mentioned. For example, flying has become something that has moved from the “few to the many”, and asking less well off people, who have only just started exploring the world, to stop flying as if they have had the same privilege as people who have seen half the world before they stopped flying is unfair. On the social justice implications of flying, see, for example, Büchs, 2017; Mattioli et al., 2023. There are related forms of activism (like the flying less movement) that perhaps should be mentioned as they form another wing to resistance against airports – see, for example, the award-winning piece in *Environmental Politics* by Katz-Rosene et al. (2024). The site here is definitely outside of the airport, but still an important part of anti-airport activism.

 I suggest that the methodology is significantly revised to improve the replicability of the research – at least regarding the search of newspaper databases. Which newspapers were searched? Why were these chosen? What were the key search terms used? Was any machine learning used? How were the articles actually analysed? Which social movements were selected to follow with secondary sources? And why were those particular four airports selected? Gatwick or Heathrow might have more interesting and useful case studies than Bristol airport, for example, for reasons of their centrality and the higher number of protest events there. The bigger London airports also have more comparability with a larger airport like Amsterdam Schipol.

 This final sentence in the methods section needs to be expanded upon: “ In the ongoing airport conflict in Southern Albania, activists provided updates about the development of the airport project and their protest events on a regular basis.” What does this mean? Who did they provide updates to? What sort of updates were they? How did the researcher analyse these updates? The methods section is also missing details of how the researcher moved from this broad range of methodologies to a typology. Was there thematic analysis using grounded theory approach? How was this carried out?

 The methods section also needs to justify the choice of 2019-2024. Was this time span chosen intentionally to capture before-and-after the pandemic dynamics? Or are there other reasons for beginning in 2019? The 2019 start misses out significant contention in the UK context, for example from Plane Stupid (see, for example Heiko Garrelts, 2014), and the Camps for Climate Action (e.g. the 2007 camp outside Heathrow airport).

 The timeframe of 2019-2024 is selected, and yet the second category of types of airport dissent refers to protests in 1973 and 1980. How do those cases fit into this research design?

- Are they reported in the source material 2019-2024? Or,
- Are they used as basic contextual material from outside of the study design? If so, why?

The results section almost begs for some comparative analysis. I understand that this is a qualitative study, yet it would still be interesting to understand whether the five different types of protest are differentially distributed across the different airports / country-cases selected. Doing so could give the paper more analytical leverage. The results section also seems to include items not connected to the research methodology (for example, in Type II Stansted is mentioned, and in Type IV, Heathrow is mentioned, and yet these are not study sites). This is not necessarily a problem, it just means that the researcher needs to more clearly articulate the methodology, explaining where and why other airport site details are included.

 I think a new sub-heading might be required when the description of Type V ends. As the paper is currently laid out, it appears that aircraft noise is being discussed under Type IV and Type V. A new subheading before the paragraph that begins “In both type IV and Type V” would help with signposting.

 The discussion of digital activism is important, but it is difficult to see how this is related to the literature review, or the overall arguments of the piece. One could say the same about the section on “the post-colonial question”. This is obviously a super-important debate. Like the section on spatial politics, it reads really well, but both would benefit from being better foregrounded in the set up of the piece (i.e. in the opening pages). The opening pages could clearly articulate the research questions and show how these require being answered by problems with or gaps in the existing knowledge base. Failure to do this means that it can be difficult to see what is the contribution of the article. It also means that the article provides several points of debate, and that it is, at present, somewhat difficult to see how they all hang together The typology, spatialities, online activism and the post-colonial question are matters that could be brought into much closer dialogue with each other throughout the paper.

 The discussion should do a better job of linking together the respective parts of the paper, and being clearer about how the paper contributes to knowledge and builds on existing research. I would recommend to remove discussion of the success and failure of protests as this seems very much beyond the scope of the present paper. Moreover, as the author admits, understanding the success of movements is mere speculation. The Discussion section should probably not introduce new material (e.g. Scientist Rebellion in Denmark); but should instead do the analytical grind of tying together the different parts of the argumentation.

 The paper mentions how airport events have intensified. Has that happened between 2019 and 2024? Can this claim be substantiated? For example, how many of the 140 protest events studies at airports in Europe took place in 2019 compared to 2024? It would be really interesting to see some sort of trend line. This would also neatly illustrate the huge dip in airport protests during the pandemic.

 I note that the conclusion seems to drift back towards climate change protests at the expense of the other four types of airport protest identified in the typology. I would suggest that this is redressed by giving more equal balance to all five types. Another, more radical, solution is to adjust the rest of the paper more significantly by zooming in specifically on the climate-related protests.

**References**

 Please refer to reference 1-4 below.

If applicable, is the statistical analysis and its interpretation appropriate?

Not applicable

Is the study design appropriate and is the work technically sound?

Partly

Is the work clearly and accurately presented and does it engage with the current literature?

Partly

Are the conclusions drawn adequately supported by the results?

Partly

Are sufficient details of methods and analysis provided to allow replication by others?

Partly

Are all the source data and materials underlying the results available?

No source data required

Reviewer Expertise:

Environmental protest, organising for change, environmental activist networks, protest surveys, environmental justice, co-creative methodologies.

I confirm that I have read this submission and believe that I have an appropriate level of expertise to state that I do not consider it to be of an acceptable scientific standard, for reasons outlined above.

## References

[1] : The role of values for voluntary reductions of holiday air travel. *Journal of Sustainable Tourism* .2017;25(2) : 10.1080/09669582.2016.1195838 234-250 10.1080/09669582.2016.1195838

[2] : Who flies but never drives? Highlighting diversity among high emitters for passenger transport in England. *Energy Research & Social Science* .2023;99: 10.1016/j.erss.2023.103057 10.1016/j.erss.2023.103057

[3] : Spiral-scaling climate action: lessons from and for the academic flying less movement. *Environmental Politics* .2024;33(2) : 10.1080/09644016.2023.2193068 259-280 10.1080/09644016.2023.2193068

[4] : Organization profile: Plane Stupid. *Routledge Handbook of the Climate Change Movement (Routledge)* .2014;

# Reviewer report: Reviewer response for version 1

**DOI:** 10.21956/openreseurope.20239.r71310

**Published:** 2026-04-20

**Authors:** Katrina Navickas

**Affiliations:** 1 University of Hertfordshire, Hatfield, England, UK

This is a wide-ranging study of 140 protest events at global airports between 2019 and 2024. It offers a typology and categorisation of different protest causes, from labour disputes to climate protests to protests about international issues using the airport as a focus. It then examines sites of protest in and outside the airport. The discussion section then raises questions about the data, though it generally has to conclude that many of the factors are variable and differ according to location. It also raises the difficulty of assessing whether protests are successful in achieving their aims. 

 The data and methods are solid and well explained. The range of airports chosen, and the balance between qualitative secondary data and direct observation is well explained. 

 The article would benefit from some wider comparative data about protests before the period of study - with the rise of environmental direct action, have airports become more targeted than previously as sites of protest? The airport's role as public space is emphasised in the introduction - how might this compare with other types of infrastructure or airspace that might be targeted by environmental or political protesters - e.g. air force bases? The typology of reasons for protest highlights labour disputes - has this form of protest become less common given that outsourcing is now more prevalent? Given that the scope of the data is global, what regional differences can be observed from the findings - e.g. are protests more likely in the Global North or in the Global South? Are there other geographical patterns that can be identified? 

 Consideration of these questions may help deepen the analysis and provide opportunities for more comparative work. But the article sets out to do what it outlines, with a solid base of data and evidence, so it does not require modification at this stage.

If applicable, is the statistical analysis and its interpretation appropriate?

Yes

Is the study design appropriate and is the work technically sound?

Yes

Is the work clearly and accurately presented and does it engage with the current literature?

Yes

Are the conclusions drawn adequately supported by the results?

Yes

Are sufficient details of methods and analysis provided to allow replication by others?

Yes

Are all the source data and materials underlying the results available?

Yes

Reviewer Expertise:

Historical geography; history of protest and social movements

I confirm that I have read this submission and believe that I have an appropriate level of expertise to confirm that it is of an acceptable scientific standard.

# Reviewer report: Reviewer response for version 1

**DOI:** 10.21956/openreseurope.20239.r69324

**Published:** 2026-04-06

**Authors:** Benjamin Bowman, Katherine A. Smith

**Affiliations:** 1 University of York, York, UK; 2 Manchester Metropolitan University, Manchester, England, UK

This is a strong, original and potentially important article which provides an important contribution through analysis of the airport as a site of political participation. As the authors explain, this is an emerging field where research is necessary, and the article provides helpful guidance to scholars who hope to undertake research in and about airports. It also gives a strong descriptive account of many protests over several decades which helps to place the question of airport protest within in its historical and spatial contexts. 

 A strength of the article lies in its descriptive accounts of the airport as a site of protest. The article includes a wide range of examples. Importantly, the authors include examples that defy simple description, reflecting - for instance - on the complex motivations of Black Lives Matter activists at London City Airport in 2016. 

 The article could be strengthened with a clearer research question, which would help guide readers to understand the approach of the authors. Similarly, while the authors describe the sources of their data, we felt there was a lack of description within the article as to the analytical methods adopted. While these methods were helpful and the article is a strong one, a more thorough understanding of analytical methods used may also help to understand and justify how the various categories within the typology outlined were reached. As things stand, and while no typology is perfect, we are left wondering around certain crossovers between categories. For example, climate change protest is a category on its own, however it also falls under type 2 (national and international affairs) as well as type 4 (externalities of the airline industry). As another example, climate change is identified as a key issue in airport protest, and we think there is an opportunity in this article or in future research to unpack climate change as a concept: this might mean, for instance, observing the difference between what Laura Pulido (1996) called mainstream environmentalism (with an emphasis on sustainability and carbon emissions) and environmental justice (more focussed on civil rights, global injustice and interconnected struggles for justice).

 The article provides a strong exploration of literature in relation to the airport. On the other hand, it does not engage as effectively with the literature surrounding protest and political participation, specifically literature on typologies of political participation (e.g.- and among others- Grasso, 2016;  Ekman and Amna, 2012), which all provide analyses of protest/ political action with differing analytical foci. A recognition of this literature, and an exploration how this paper relates to the protest/ participation literature would help to cement more clearly how this relates to existing work in this field. 

 A couple of notes in relation to the language adopted, the article at times refers in unclear  albeit somewhat pejorative terms to actors such as “irregular” migrants or “impoverished countries”. While this may be unintentional, and the authors do make use of inverted commas, our suggestion would be to describe these actors in more neutral terms such as “countries with high levels of poverty”/ a more descriptive and neutral wording of “irregular”. 

 Overall, the article is very interesting and will no doubt provide an important contribution. However, we encourage the author to address these issues before indexing in order to ensure a stronger contribution to the different literatures in which this research sits.

If applicable, is the statistical analysis and its interpretation appropriate?

Not applicable

Is the study design appropriate and is the work technically sound?

Yes

Is the work clearly and accurately presented and does it engage with the current literature?

Yes

Are the conclusions drawn adequately supported by the results?

Yes

Are sufficient details of methods and analysis provided to allow replication by others?

Yes

Are all the source data and materials underlying the results available?

Yes

Reviewer Expertise:

NA

We confirm that we have read this submission and believe that we have an appropriate level of expertise to confirm that it is of an acceptable scientific standard, however we have significant reservations, as outlined above.

# Reviewer report: Reviewer response for version 1

**DOI:** 10.21956/openreseurope.20239.r69322

**Published:** 2026-03-27

**Authors:** Alice Mattoni

**Affiliations:** 1 Department of Political and Social Sciences, University of Bologna, Bologna, Italy

The article explores protest events concerning airports through a historical-spatial analysis of 140 protest events that occurred in Europe between 2019 and 2024. In so doing, the article addresses the issue of spatiality in social movements and, more specifically, mobilizations. The topic is certainly valuable, and it does provide relevant information on a type of protest, the one concerning airports, that is not that explored in social movement studies. Overall, the article is well written, and its arguments are clear and convincing. There is, however, some room for improvement, and below I offer some suggestions in this direction.

 In a couple of cases, the article would benefit from more conceptual clarity. In particular, I think that the author should explain better what prefigurative legitimacy is, in general, but also how it might be relevant in the framework of the present study. More importantly, the author seems to consider protest events as political communication. I think this take on protest events might be revised by considering them not as political communication in themselves, but as forms of collective action with a communicative dimension, since activists organize and perform them to communicate something to different audiences. In this regard, the classic work by Charles Tilly on repertoire of protest might be useful.

 Concerning methods, I ask the author to include more detailed information on how the dataset, including the 140 protest events, has been built, for instance, concerning the newspapers and news sampling, and also, what type of information it includes exactly. Beyond this, it is not clear whether there is one dataset containing information on the 140 protest events and another dataset containing the raw newspaper articles. This should be clarified. Importantly, a brief discussion of the limitations of using newspaper articles to compile the dataset, including the 140 protest events, would be welcome. Finally, the article speaks about a historical-spatial analysis, but then how this analysis was conducted, exactly, and what it entailed methodologically speaking, is not there in the methods section. I urge the author to be more transparent concerning the data analysis to strengthen their work. 

 The typology built from the 140 protest events and the related discussion is convincing and valuable. However, one question remains to be answered: what does this analysis and its findings tell us about how space and place are treated in social movement studies? That is to say, beyond the important work done here in terms of providing a detailed description of protest events concerning airports, what is it that we learn in connection to the broader topic of spatiality in social movements and their mobilizations? I would welcome a discussion of this aspect in the Discussion section and, also, in the Conclusions. The interesting question here is: what do we learn from the study you produced that is useful for social movement studies and advances them? What do you add to the debate on spatiality in social movement studies? Addressing this question would also require some revision in the Introduction and the Literature Review sections of the paper.

 Finally, the section on the post-colonial question is also relevant and informative, but, as it stands, it is quite detached from the rest of the article. My suggestion would be to condense it and include it in the Conclusions, so they can offer the reader critical remarks on problems and protests related to airports in other countries in the Global South, and explain their relevance as case studies to enrich and further expand insights on spatiality in social movements.

If applicable, is the statistical analysis and its interpretation appropriate?

Not applicable

Is the study design appropriate and is the work technically sound?

Yes

Is the work clearly and accurately presented and does it engage with the current literature?

Yes

Are the conclusions drawn adequately supported by the results?

Yes

Are sufficient details of methods and analysis provided to allow replication by others?

Partly

Are all the source data and materials underlying the results available?

Yes

Reviewer Expertise:

social movement studies, qualitative research design, communication and media studies

I confirm that I have read this submission and believe that I have an appropriate level of expertise to confirm that it is of an acceptable scientific standard, however I have significant reservations, as outlined above.

# Reviewer report: Reviewer response for version 1

**DOI:** 10.21956/openreseurope.20239.r69320

**Published:** 2026-03-11

**Authors:** Pamela Oliver

**Affiliations:** 1 University of Wisconsin –Madison, Madison, Wisconsin, USA

This paper is empirically solid. It provides details about protests at airports including both a qualitative review of historical cases and cases outside of Europe drawn from secondary sources and news accounts and a descriptive quantitative summary of 140 European airport protests between 2019 and 2024. There is also mention of participant observation of protests at airports in two countries although this plays little role in the manuscript. I found the paper to be full of well-documented information about airport protests. It can stand on its own as providing comprehensive information about its stated topic.

 The literature review appears to provide a comprehensive account of everything that has been written about protests at airports. As noted below, however, the literature review is silent about how airport protests might compare with protests about similar issues at other locales or about how airports compare to other protest locales. The different sections of the literature review are linked by their mention of airports but not by other theoretical concerns. As just one example, airports today as sites of protest are probably usefully compared to railroads (or railroad stations) and to the ships, docks, canals etc. of water transport in prior eras.

 The paper offers a 5-category typology of the reasons for (or issues of) protests at airports: worker struggles, national or international issues unrelated to the airport itself, services provided by airports, airport externalities, climate effects of air travel.  Each of these “types” implies different non-airport comparisons.

 Another section discusses the diverse tactics of airport protests and the different locations within the airport where the protest might occur but makes no attempt to cross-classify this location dimension with the issue-type dimension.

 A final section on the postcolonial dimension again contains useful qualitative descriptions of airport protests outside Europe but seems disconnected from the rest of the paper.

 I believe the paper stands on its own as an empirical contribution providing a good account of what is known about airport protests even as it is somewhat unsatisfactory as a contribution to broader cumulative social science research and theory.

 A wider theoretical-empirical net would connect some of these airport protests to protests at other kinds of locations, depending upon the issue type. Each of the five types of issues undergirding airport protests would logically involve comparisons with similar protests at other sites. The author notes that protests about the climate impacts of air travel occur at places other than airports.

 Worker protests obviously happen at all kinds of workplaces. Protests by airport workers would usefully be compared to protests by railway workers or dock or marine workers. All of these are key transportation institutions that are vulnerable to protest and include workers who travel widely as well as those who remain in one place.

 Protests about issues unrelated to airports can usefully be contrasted with protests about similar issues in other locations. Protests about airport services can be usefully contrasted with protests about services by other industries.

 Protests about the external effects of airports on the surrounding communities are most usefully compared with other protests about the placement of noxious industries and the displacement of residents for non-residential development projects.

 Overall, the paper suggests many follow-up studies that would locate airport protests of a particular type in a larger frame, or that would highlight the specific consequences of protesting at an airport rather than elsewhere.

 That said, this document still stands on its own for accomplishing what it set out to do, which is to provide a comprehensive account of what is known about protests at airports. I do not see any value in attempting to revise this document except possibly for acknowledging the need for future research to find points of comparison between protests at airports and protests at other kinds of sites.

If applicable, is the statistical analysis and its interpretation appropriate?

Not applicable

Is the study design appropriate and is the work technically sound?

Yes

Is the work clearly and accurately presented and does it engage with the current literature?

Yes

Are the conclusions drawn adequately supported by the results?

Yes

Are sufficient details of methods and analysis provided to allow replication by others?

Not applicable

Are all the source data and materials underlying the results available?

Yes

Reviewer Expertise:

I am a sociologist whose main field of study is protests and social movements and news media coverage of protests and social movements.

I confirm that I have read this submission and believe that I have an appropriate level of expertise to confirm that it is of an acceptable scientific standard.
